# Effects of perioperative pelvic floor muscle training on early recovery of urinary continence and erectile function in men undergoing radical prostatectomy: a randomized clinical trial

**DOI:** 10.1590/S1677-5538.IBJU.2019.0238

**Published:** 2019-12-17

**Authors:** Gislano Heverton Soares de Lira, Alexandre Fornari, Luiz Felipe Cardoso, Magda Aranchipe, Carmem Kretiska, Ernani Luis Rhoden

**Affiliations:** 1 Universidade Federal de Ciências da Saúde de Porto Alegre (UFCSPA), Porto Alegre, RS, Brasil;; 2 Unidade de Disfunção Miccional, Santa Casa de Misericòrdia de Porto Alegre, Porto Alegre, RS, Brasil;; 3 Departamento de Urologia, UFCSPA, Porto Alegre, RS, Brasil

**Keywords:** Prostatic Neoplasms, Urinary Incontinence, Erectile Dysfunction, Quality of Life

## Abstract

**Aims:**

Radical prostatectomy (RP) can result in urinary incontinence (UI) and erectile dysfunction (ED), which negatively impact quality of life (QoL). This study aimed to evaluate the effects of a perioperative pelvic floor muscle training (PFMT) program versus usual care on early recovery of urinary continence and erectile function after RP.

**Materials and Methods:**

Of 59 eligible men, 31 were randomly allocated into 2 groups: Group 1 (Control, N=15) received usual post-RP care; and Group 2 (Physical therapy, N=16) received two pre-RP physical therapist-guided PFMT sessions, including exercises and electromyographic biofeedback, and verbal and written instructions to continue PFMT until RP, which was then resumed after urethral catheter removal. The International Consultation on Incontinence Questionnaire - Short Form (ICIQ-SF) and the 5-item version of the International Index of Erectile Function (IIEF-5) questionnaire were used to evaluate UI and ED, respectively.

**Results:**

Demographic characteristics were similar in both groups. Three months after RP, the UI rate was 72.7% and 70.0% in Groups 1 and 2, respectively (P >0.05). The severity and frequency of UI and its impact on QoL were evaluated by the ICIQ-Short Form, with scores of 6.9±6.26 in Group 1 and 7.0±5.12 in Group 2 (P >0.05). The IIEF-5 scores were similar in Groups 1 and 2 (5.73±7.43 vs. 6.70±6.68, respectively) (P >0.05).

**Conclusion:**

Our pre-RP protocol of two physical therapist-assisted sessions of PFMT plus instructions did not significantly improve urinary continence or erectile function at 3 months after RP.

## INTRODUCTION

Prostate cancer is a common malignancy in older men. Surgical treatment involving the removal of the prostate may result in temporary or permanent erectile dysfunction (ED) and urinary incontinence (UI), with a major impact on quality of life (QoL) (1, 2).

Despite advances in surgical techniques and knowledge of pelvic anatomy, the prevalence of post-prostatectomy UI ranges from 1 to 87%, depending on the definition, period of evaluation, surgical technique, preoperative condition of the patient, and the evaluation tool (3). ED affects 26 to 100% of patients after radical prostatectomy (RP), and the major cause is known to be injury to the neurovascular bundles (4, 5). However, other mechanisms, including arterial trauma and structural alterations within the corpora cavernosa smooth muscle, may affect erectile function after RP (4). The negative postoperative effects of RP on erectile function and the QoL of affected men and their sexual partners may persist longer than the concern about the effectiveness of cancer treatment (5).

Pelvic floor muscle training (PFMT) is one of the recommended techniques for the prevention, treatment and rehabilitation of RP-related complications. However, data in the literature are scarce, and some results are controversial in the context of both UI and ED (6–16). There is a lack of standardized treatment protocols, especially regarding preoperative PFMT and its benefits (17).

The current trial was therefore designed to evaluate the effects of a PFMT protocol, including two preoperative physical therapist-guided sessions as well as verbal and written instructions to continue the exercises after surgery, on the urinary continence and erectile function of men undergoing RP.

## MATERIALS AND METHODS

This was a single-center, prospective, randomized, parallel-group (1:^1^), controlled trial of patients undergoing open retropubic RP for localized prostate cancer. Eligible participants were all patients aged 45 to 75 years with prostate adenocarcinoma who were candidates for RP at our institution from March 2013 to December 2014. Exclusion criteria were previous pelvic radiotherapy, presence of neurological disorders, laparoscopic RP, previous transurethral resection of the prostate, presence of incontinence, or inability to perform pelvic floor exercises.

At baseline, a complete medical history and physical examination were performed on all patients (including measurements of weight, height, and abdominal and hip circumferences). Patients also answered the 5-item version of the International Index of Erectile Function (IIEF-5) questionnaire, which classifies ED into five categories based on the scores obtained: severe (1–7); moderate (8–11); moderate to mild (12–16); mild (17–21); or no ED (22–24). In the same evaluation, electromyographic recordings of the pelvic floor were obtained using the Miotool Uro device (Miotec^®^, Brazil), including the average and maximum values of electromyographic activity at rest and during rapid and sustained contraction of the pelvic floor.

A computer-generated list of random numbers (WinPepi, version 2.62) was used to allocate patients into one of two groups: Control Group, which included men who received only usual post- RP care, and Physical Therapy Group, which included men who received two preoperative PFMT sessions guided by a physical therapist (MA), including exercises and electromyographic biofeedback (to ensure that the patient had learned how to perform the exercises correctly), and were instructed to perform the exercises throughout the preoperative period and to resume them immediately after removal of the urethral catheter. Patients exercised three times a day at progressively higher intensities. Blinding of participants was not possible.

All patients were re-evaluated 3 months after RP, when electromyographic recordings were again obtained by another physical therapist (CK) who was blinded to group assignment, including all patterns and using the same electromyography device. Patients also answered the IIEF-5 questionnaire and the International Consultation on Incontinence Questionnaire -Short Form (ICIQ-SF), a validated questionnaire designed to evaluate UI. By analyzing the ICIQ--SF scores, we can determine the intensity and frequency of post-prostatectomy UI, as well as how much it affects QoL. All patients were asked about urine loss in any amount. UI was defined as the patient's perception of loss of at least a few drops of urine.

The primary endpoint was the between-group difference in UI and ED as measured by the respective questionnaires. The secondary endpoint was the between-group difference in the electromyographic values obtained at the 3-month postoperative evaluation.

The sample size was calculated using Win-Pepi, version 2.62. To detect a 75% lower UI rate (loss of drops of urine) in the Physical Therapy Group than in the Control Group, with a power of 80%, a minimum sample size of 16 patients per group was necessary. Patients were included in the study until December 2014, which was the end of the recruitment period. Quantitative variables with symmetric distribution were expressed as mean and standard deviation (mean±SD) and compared using Student's t test for independent samples. Categorical variables were analyzed using the chisquare test or Fisher's exact test. The McNemar test was used to compare categorical variables within groups between the preoperative and postoperative periods. The Mann-Whitney U test was used for variables with asymmetric distribution. For correlation analysis, the Pearson correlation coefficient was used. The significance level was set at 5%.

The trial protocol was approved by the Research Ethics Committee of the institution, and written informed consent was obtained from all individual participants before enrollment. The study followed the CONSORT guidelines for the reporting of randomized controlled trials. The trial is registered at the Brazilian Clinical Trials Registry platform (ReBEC).

## RESULTS

**Figure 1:**
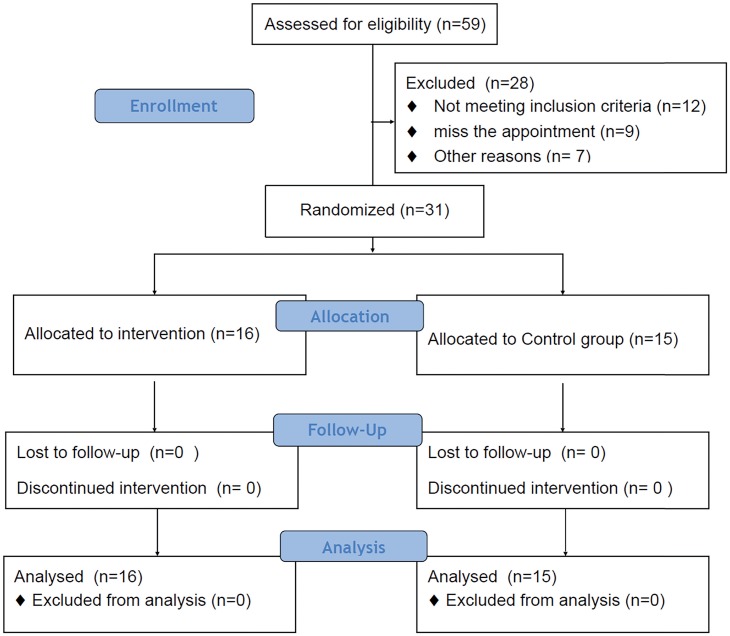
Consort flowchart.

Of 59 eligible men with prostate adenocarcinoma treated at our institution during the study period, 31 met the inclusion criteria and were randomly allocated into one of the two study groups (Figure-1). All randomized patients completed the study (3-month post-prostatectomy evaluation) and were evaluated for the primary and secondary endpoints.

The general characteristics and anthropometric and clinical data of the study population are presented in Table-1. All of these characteristics were similar at baseline in both groups. Time (in days) to urethral catheter removal after RP was also similar in both groups (12.6±4.81 days in the Control Group vs. 12.9±2.37 days in the Physical Therapy Group, P=0.862).

**Table 1:** Baseline general characteristics of men submitted (Physical Therapy Group) or not submitted (Control Group) to pelvic floor muscle training before and after radical prostatectomy.

	Group		
Characteristics*	Control	Physical Therapy	P value**
Age (years)	63.53±7.62	67.3±5.63	0.126
Weight (kg)	83.8±19.63	75.5±8.03	0.145
BMI (kg/m^2^)	28.34±5.64	26.2±3.0	0.220
Abdominal circumference (cm)	105.61±18.80	97.5±5.97	0.162
Hip circumference (cm)	103.61±11.16	97.3±4.4	0.107
Preoperative PSA (ng/dL)	9.20±4.65	14.1±11.19	0.126
Preoperative IIEF-5 score	16.3±7.64	16.7±6.65	0.872

**BMI** = body mass index; **IIEF-5** = 5-item version of the International Index of Erectile Function; **PSA** = prostate specific antigen.

* All values are expressed as mean ± standard deviation.

** Student's t test for independent samples.

The Gleason score and pathological stage of the surgical specimens were divided into categories, and patients in the Physical Therapy Group tended to have tumors with more aggressive features (i.e., a higher Gleason score and pathological stage). However, these variables and the positive margin rate were statistically similar in both groups (P >0.05) (Table-2).

**Table 2:** Tumor stage and pathological features of prostate cancer in patients undergoing radical prostatectomy and submitted (Physical Therapy Group) or not submitted (Control Group) to pelvic floor muscle training for recovery of urinary continence and erectile function.

		Group		
Characteristics		Control	Physical Therapy	P value*
Prostate size (g)**		42.1±9.09	47.7±12.58	0.186
Pathological tumor stage (%)***				
	≤ pT2c	71.4	35.7	0.130
	pT3a	21.4	21.4	1.000
	≥ pT3b	7.1	42.9	0.070
**Gleason score (%)**				
	≤6	21.4	0.0	0.222
	7	71.4	57.1	0.693
	≥8	7.1	42.9	0.070
Positive margin (%)		14.3	42.9	0.209

* Chi-square test with Yates correction or Fisher's exact test for categorical variables; P<0.05.

** Values are expressed as mean ± standard deviation.

*** Pathological staging (pT) according to the TNM system of the American Joint Committee on Cancer (AJCC).

Three months after RP, the UI rate was 72.7% in the Control Group and 70.0% in the Physical Therapy Group (P >0.05). There was no significant difference in ICIQ-SF scores between the Control Group (6.9±6.26) and Physical Therapy Group (7.0±5.12) (P=0.97).

Regarding erectile function, at baseline, patients were classified as having moderate to mild ED according to the IIEF-5 scores, with no between-group difference (Table-1). Three months after surgery, there was also no significant difference between the two groups, although there was a tendency toward lower scores in the Control Group (58.3% [5.73±7.43]) than in the Physical Therapy Group (52.7% [6.70±6.68]) (P=0.745).

The average and maximum values of electromyographic activity recorded at rest and during rapid contraction of the pelvic floor and sustained contraction of the external anal sphincter before and after RP in both groups are presented in Table-3. Electromyographic activity was similar in the two groups at baseline and at the 3-month post-RP evaluation.

**Table 3:** Electromyographic activity recorded at rest and during rapid and sustained contraction of the external anal sphincter of men submitted (Physical Therapy Group) or not submitted (Control Group) to pelvic floor muscle training before and after radical prostatectomy (RP).

		Pre RP			Post RP		
Electromyographic activity a,b		Control *N=15*	Physio *N=16*	P value	Control *N=15*	Physio *N=16*	*P* value*
	At rest	0.64±0.35	0.77±0.15	0.184	0.88±0.14	0.84±0.16	0.466
	Average	0.94±0.34	1.06±0.28	0.291	1.12±0.12	1.05±0.27	0.364
	Maximum	0.64±0.35	0.77±0.15	0.184	0.88±0.14	0.84±0.16	0.466
**Rapid contraction**							
	Average	0.98±0.29	0.99±0.19	0.919	1.00±0.12	0.98±0.18	0.720
	Maximum	1.59±0.25	1.45±0.23	0.115	1.50±0.20	1.49±0.25	0.903
**Sustained contraction**							
	Average	1.16±0.46	1.23±0.27	0.606	1.26±0.17	1.24±0.27	0.808
	Maximum	1.42±0.41	1.43±0.29	0.938	1.49±0.16	1.45±0.27	0.623

a All values are expressed as mean ± standard deviation.

b The data were log transformed to normalize and standardize the distribution of electromyographic activity data.

* Student's t test for paired samples; P<0.05.

## DISCUSSION

In the current study, pelvic floor physical therapy was proposed both to prevent and treat surgical complications and to rehabilitate UI and ED in patients with prostate cancer undergoing RP. For this purpose, we developed a low-cost and easy-to-perform physical therapy program in an attempt to investigate potential positive effects on the recovery of these side effects of RP, while perhaps improving the QoL of the affected individuals. Nevertheless, based on the results of the current study, we were unable to demonstrate significant positive effects in an early (3-month) evaluation by using a simple approach based on the patient's answers to specific UI and ED questionnaires.

In a study with similar methodology, Parekh et al. (8) evaluated 38 men and reported that the group of patients submitted to pre- and post-RP pelvic floor exercises plus instructions to continue the exercises in the postoperative period twice a day regained urinary continence earlier than patients in the group without formal physical therapy treatment (control group). However, in that study, the number of sessions guided by a physical therapist was higher than that used in the current study. Another interesting study comparing preoperative and postoperative PFMT plus biofeedback (group A) with preoperative PFMT alone (group B) found a continence rate of 6.6% in both groups at 1-month follow-up and of 33.3% in group A vs. 26.6% in group B at 3 months after RP (15), results that are similar to ours (urinary continence of 30% in the Physical Therapy Group vs. 27.3% in the Control Group).

The outcomes related to UI and QoL observed in the current study were similar to those published in a meta-analysis (18), in which preoperative PFMT did not contribute to improving the recovery of urinary continence at 3, 6 and 10 months after RP and had no conclusive positive effects on the QoL of patients treated with RP. Furthermore, in a study of the effect of postoperative PFMT for up to 1 year after RP, despite the improvement observed in the urinary continence of patients undergoing physical therapy, there was no improvement in the parameters related to QoL (19). It is important to note that our protocol with only two physical therapist-guided sessions did not improve electromyographic parameters (that could be related to muscle strength), not even in the Physical Therapy Group; therefore, this finding may explain why there was no improvement in continence rates. Nowadays, we discontinued this protocol and currently use protocols with at least 6 sessions of PFMT and close monitoring of the patient by the physical therapist.

Most of the evidence on the improvement of erectile function with the use of pelvic floor exercises in men with ED is associated with conditions other than RP (20, 21). In order to evaluate the potential beneficial effects of such interventions, Prota et al. (22) studied 52 patients undergoing RP, with a 12-month follow-up, and showed that PFMT associated with biofeedback initiated after urethral catheter removal had a positive impact on the recovery of erectile function (absolute risk reduction of 34.6%; 95% confidence interval: 3.8-64%). Similar to this study, in which the average IIEF-5 score in both groups was lower than 10 at 3 months postoperatively, we also observed a significant reduction in IIEF-5 scores 3 months after surgery (5.73±7.43 in the Control Group vs. 6.70±6.68 in the Physical Therapy Group). The early assessment of ED may be one of the reasons why we have not observed any benefit from PFMT in the recovery of erectile function.

An interesting study using electromyography in the diagnosis and treatment of stress UI in women showed differences in the peak values for rapid contraction at rest and during exercise (14.56 vs. 21.67 microvolts in incontinent vs. healthy women, respectively) (23). Applying this concept to individuals treated with RP, as in the current study, based on the electromyographic activity obtained through electromyographic biofeedback with an endoanal probe, no statistically significant changes or differences were observed between the two groups at rest and during rapid or sustained contraction. Therefore, it remains unclear whether a PFMT program applied for 3 months without close supervision and feedback from a physical therapist can strengthen the pelvic muscles and/or recover possible post-RP muscle damage and its impact on urinary continence recovery.

This study has some limitations that should be considered. First, the sample size was relatively small, and we did not achieve the required number of 16 patients in the Control Group (only 15 patients were included in this group). Second, subtle differences in the results might have been observed if we had adopted objective measures of UI, such as the PAD test (1 or 24 hours). Third, it is unknown how much urine loss is significant in men with post-RP UI and how this impacts the patient's perception and answers. Fourth, few studies have investigated the effect of education strategies (e.g., information guides for patients), specifically following the delivery of preoperative treatment (24). Finally, and perhaps most importantly, most of the protocols that have been evaluated and employed in the literature include a larger number of PFMT sessions and closer monitoring of the patient by the physical therapist, which may have directly influenced the results. However, the main strengths of the current study include its randomized design, with similar treatment and control groups, a strict study protocol, in which all randomized patients were analyzed, and a team of blinded evaluators.

## CONCLUSIONS

The protocol of two supervised PFMT sessions with biofeedback in the preoperative period plus verbal and written instructions to continue the exercises after surgery did not exert a sufficient effect to improve continence rates or erectile function in an early (3-month) evaluation after open retropubic RP. However, new protocols with a higher level of intervention and close monitoring of the patient by a trained physical therapist should be investigated to clarify the role of perioperative PFMT in the recovery of urinary continence and erectile function after RP.

### Compliance with Ethical Standards

The trial is registered at the Brazilian Clinical Trials Registry plataform (ReBEC) number RBR-7s6khg
